# Markers of Collagen Remodeling Detect Clinically Significant Fibrosis in Chronic Hepatitis C Patients

**DOI:** 10.1371/journal.pone.0137302

**Published:** 2015-09-25

**Authors:** Mette J. Nielsen, Konstantin Kazankov, Diana J. Leeming, Morten A. Karsdal, Aleksander Krag, Francisco Barrera, Duncan McLeod, Jacob George, Henning Grønbæk

**Affiliations:** 1 Nordic Bioscience A/S, Fibrosis Biology and Biomarkers, Herlev, Denmark; 2 Department of Gastroenterology and Hepatology, Odense University Hospital, University of Southern Denmark, Faculty of Health Science, Odense, Denmark; 3 Department of Hepatology and Gastroenterology, Aarhus University Hospital, Aarhus, Denmark; 4 Storr Liver Unit, Westmead Millennium Institute, Westmead Hospital and University of Sydney, Sydney, Australia; 5 Department of Anatomical Pathology, Institute of Clinical Pathology and Medical Research, Westmead Hospital, Sydney, Australia; University of Navarra School of Medicine and Center for Applied Medical Research (CIMA), SPAIN

## Abstract

**Background and Aim:**

Detection of advanced fibrosis (Metavir F≥3) is important to identify patients with a high urgency of antiviral treatments vs. those whose treatment could be deferred (F≤2). The aim was to assess the diagnostic value of novel serological extracellular matrix protein fragments as potential biomarkers for clinically significant and advanced fibrosis.

**Methods:**

Specific protein fragments of matrix metalloprotease degraded type I, III, IV and VI collagen (C1M, C3M, C4M, C6M) and type III and IV collagen formation (Pro-C3 and P4NP7S) were assessed in plasma from 403 chronic hepatitis C patients by specific ELISAs. Patients were stratified according to Metavir Fibrosis stage; F0 (n = 46), F1 (n = 161), F2 (n = 95), F3 (n = 44) and F4 (n = 33) based on liver biopsy.

**Results:**

Pro-C3 was significantly elevated in patients with significant fibrosis (≥F2) compared to F0-F1 (p<0.05), while the markers C3M, C4M, C6M and P4NP7S were significantly elevated in patients with advanced fibrosis (≥F3) compared to F0-F2 (p<0.05). C1M showed no difference between fibrosis stages. Using Receiver Operating Characteristics analysis, the best marker for detecting ≥F2 and ≥F3 was Pro-C3 with AUC = 0.75 and AUC = 0.86. Combination of Pro-C3 and C4M with age, BMI and gender in a multiple ordered logistic regression model improved the diagnostic value for detecting ≥F2 and ≥F3 with AUC = 0.80 and AUC = 0.88.

**Conclusion:**

The Pro-C3 protein fragment provided clinically relevant diagnostic accuracy as a single marker of liver fibrosis. A model combining Pro-C3 and C4M along with patient’s age, body mass index and gender increased the diagnostic power for identifying clinically significant fibrosis.

## Introduction

Chronic hepatitis C (HCV) affects approximately 170 million people worldwide, and represents a major healthcare problem due to the high risk for fibrosis development and consequently cirrhosis and hepatocellular carcinoma [1]. Liver biopsy is the gold standard to stage liver fibrosis; however, limitations such as sampling error and observer variability as well as potential complications limit its use [2, 3]. Conversely, accurate fibrosis staging is important to decide the timing of antiviral therapy and to identify those at high risk of disease progression. Thus, there remains an unmet need for more reliable non-invasive markers to stage liver fibrosis.

Various candidates have been proposed as biomarkers for liver fibrosis either alone or in combination, including FibroTest [4], HepaScore [5], FIBROSpect [6], and recently sCD163, a macrophage activation marker [7, 8]. These serological tests are principally based on liver tests or inflammatory proteins, rather than structural markers such as hyaluronic acid (HA), N-terminal propeptide of type III collagen (PIIINP), or in combination as the Enhanced Liver Fibrosis (ELF) test [9].

Structural changes in the extracellular matrix (ECM) as a reflection of matrix turnover are however especially attractive to stage liver fibrosis as the remodeling results in release of protein products related to both synthesis and degradation of the ECM [10–13]. Indeed, neo-epitope based biomarkers represent a unique fingerprint of proteolytic cleavage of the ECM where measurement of different sub-pools of the same protein may provide different information. We recently developed a panel of serological markers for fibrosis using this fingerprint technology, which represent particular types of ECM remodeling in fibrotic diseases of varying etiology [14–20].

We hypothesized that measurement of both degradation and formation markers of ECM remodeling could provide an alternative to existing non-invasive serum markers to assess disease stage in a large cross-sectional study of patients with chronic HCV. The primary aim was to evaluate a panel of Protein Fingerprint markers to identify patients with a high urgency of antiviral treatments (Metavir Fibrosis ≥F3) and those whose treatment could be deferred (Metavir Fibrosis ≥F2) in patients with HCV compared to the traditional markers alanine aminotransferase (ALT) and aspartate aminotransferase (AST), and the algorithms FIB-4 and AST to platelet ratio index (APRI). A secondary aim was to evaluate whether the Protein Fingerprint markers could differentiate patients with fibrosis (≥F1) from those without. The study found that a marker of true type III collagen formation (Pro-C3) provided clinically relevant diagnostic accuracy as a single marker of liver fibrosis. A model combining both formation and degradation markers along with patient’s age, body mass index and gender increased the diagnostic power for identifying clinically significant fibrosis.

## Patients and Methods

In a cross-sectional study-design we included chronic HCV infected patients referred to Westmead Hospital, Westmead, Australia, between July 1991 and August 2010 for the evaluation of HCV infection. Patient baseline characteristics have been reported as part of a recently published study [7]. HCV infection was diagnosed by the presence of anti-HCV antibodies (Manolisa anti-HCV; Sanofi Diagnostics Pasteur, Marnes-la-Coquette, France) and viral RNA detected by polymerase chain reaction (PCR) (Amplicor HCV, Roche Diagnostics, Branchburg, NJ). A second generation reverse hybridization line probe was used for hepatitis C genotyping (Inno-Lipa HCV II, Innogenetics, Zwjindrecht, Belgium). None of the patients received antiviral therapy prior to inclusion. Basic demographics including age, gender, ethnicity, waist circumference, height, and weight were obtained at the time of biopsy.

Liver biopsy was performed for assessment of severity of inflammation and fibrosis. The stained biopsies were evaluated by one expert pathologist (DM) blinded to patient clinical characteristics and serum measurements and scored according to the Metavir Scoring system [21]: F0, no fibrosis; F1, portal fibrosis alone; F2, portal fibrosis with rare septae; F3, portal fibrosis with many septae; F4, cirrhosis. The presence of F2, F3 or F4 stages was referred to as significant fibrosis, while the presence of F3 or F4 stages were referred to as advanced fibrosis. All patients classified as Metavir F4 has compensated disease. All biopsies had a minimum of 11 portal tracts, and 38 inadequate biopsies were excluded, thus 403 patients were included in the study. Simultaneously, fasting blood samples were drawn for routine biochemical tests assessed by standard methods and assays, and stored at -80°C for future research. Biochemical tests included ALT, AST, alkaline phosphatase (ALP), γ-glutamyltransferase (GGT), total bilirubin, serum albumin, hemoglobin, fasting glucose and insulin, platelet and leucocyte count, international normalized ratio (INR), prothrombin time, triglycerides and cholesterol. All patients signed an informed consent form in accordance with the Helsinki Declaration. The acquisition, storage, and use of blood samples were approved by the Sydney West Area Health Service ethics committee. According to Danish legislation, ethical approval is not required when measuring biochemical markers in previously collected samples. Hence there was no ethical approval for this particular study of the Protein Fingerprint markers in these blood samples.

APRI and FIB-4 were calculated as previously described [22, 23]: APRI = (AST (IU/L)/upper normal limit)x100/platelet count (10^9^/L) and FIB-4 = age (years) x AST (UI/L)/(platelet count (10^9^/L) x (ALT (IU/L)^½^). Body mass index (BMI) was calculated as BMI = (weight (kg)/height (m)^2^).

### Protein Fingerprint marker assessment

The Protein Fingerprint markers of matrix metalloproteinase (MMP) degraded type I, III, IV and VI collagen (C1M, C3M, C4M, C6M), and type III and IV collagen formation (Pro-C3 and P4NP7S) were assessed in plasma from 403 HCV patients as previously described [14, 16, 17, 19, 20, 24]. Briefly, 96-well pre-coated streptavidin plates (Roche Diagnostics, Mannheum, Germany) were coated with the appropriate biotinylated synthetic peptides and incubated for 30 minutes at 20°C. Twenty μL of standard peptide or pre-diluted sample were added to appropriate wells, followed by peroxidase-conjugated specific monoclonal antibodies and incubated for 1 hour or overnight at 20°C or 4°C, respectively. Finally, tetramethylbenzinidine (TMB) (cat.438OH, Kem-En-Tec Diagnostics, Taastrup, Denmark) was added, and the plates were incubated for 15 minutes at 20°C in darkness. All the above incubation steps included shaking at 300 rpm. After each incubation step, the plate was washed five times in washing buffer (20 mM Tris, 50 mM NaCl, pH 7.2). The TMB reaction was stopped by adding 0.18 M H_2_SO_4_ as stopping solution and measured at 450 nm with 650 nm as reference. A calibration curve was plotted using a 4-parametric mathematical fit model.

### Statistical analysis

Data were logarithmically transformed for normality and symmetry of variance. Comparisons between the mean marker levels were performed using one-way analysis of variance (ANOVA) test with Tukey’s multiple comparisons test using each group as fixed factor. The correlation between individual markers and Metavir Fibrosis scores was analyzed by Spearman’s rank correlation. We performed multiple ordered logistic regression analyses with the Metavir Fibrosis score as dependent variable and protein fingerprint markers as well as other continuous variables as independent variables. In Model 1 we hypothesized that combination of structural markers and liver function markers would improve the liver fibrosis staging. Thus we included the protein fingerprint markers, AST, and ALT using stepwise elimination with a significance level of 0.1 as cut-off. In Model 2 we investigated the impact of demographic risk factors in combination with protein fingerprint markers for liver fibrosis. Based on a multiple ordered logistic regression model with Metavir Fibrosis score as dependent variable and protein fingerprint markers as independent variables using stepwise elimination with a significance level of 0.1, we selected the protein fingerprint markers most strongly associated with fibrosis. Thereafter we included age, gender, and BMI with direct entry of all variables. Thus Model 2 included Pro-C3, C4M, age, gender, and BMI. The diagnostic power of individual markers and combination models was investigated by the area under the receiver-operating characteristics (ROC) curve (AUC) with 95% confidence interval (CI). Sensitivity and specificity were determined for appropriate cut-off values based on the ROC curves. Pair-wise comparison of ROC curves were evaluated using the method of Delong et al [25].

Data are shown as geometric mean with 95% CI unless otherwise stated. P-values <5% were considered significant. The multiple ordinal logistic regression models were calculated in STATA version 12.0 (StataCorp LP, TX, USA). All other statistical analyses were calculated in MedCalc version 12 (MedCalc Software, Ostend, Belgium). Graphs were designed using GraphPad Prism version 5 (GraphPad Software, Inc., CA, USA).

## Results

### Patient characteristics

Patient characteristics are shown in Table 1. Patients were divided in five groups according to their Metavir Fibrosis stage (F0-F4); F0 n = 47, F1 n = 167, F2 n = 107, F3 n = 45 and F4 n = 35. The majority of the patients were male (62%) and Caucasian (90%) with a mean age of 43.5 years and a mean BMI of 26.2 kg/m^2^ with no significant differences between Metavir F stages. We observed a significant difference in ALT and AST levels with increasing severity of fibrosis (p<0.001).

**Table 1:** Patient demographics and clinical characteristics stratified according to Metavir F stages (F0-F4).

	Metavir F0		Metavir F1		Metavir F2		Metavir F3		Metavir F4		ANOVA
Parameters	*n*	Mean [95% CI] or *n* (%)	*n*	Mean [95% CI] or *n* (%)	*n*	Mean [95% CI] or *n* (%)	*n*	Mean [95% CI] or *n* (%)	*n*	Mean [95% CI] or *n* (%)	*p*-value
**Male sex**	*31*	60%	*99*	60%	*80*	68%	*36*	73%	*27*	69%	0.113
**Age, yr**	*52*	36.9 [34.1–39.6]	*175*	41.4 [39.8–42.9]	*117*	44.3 [42.7–45.8]	*49*	47.1 [44.4–49.8]	*39*	48.8[46.6–51.1]	<0.001
**BMI (kg/m** ^**2**^ **)**	*51*	26.3 [24.9–27.7]	*167*	26.1 [25.3–26.9]	*105*	27.8 [26.7–28.9]	*47*	28.3 [27.0–29.6]	*36*	27.1 [25.7–28.4]	0.020
**AST (IU/L)**	*46*	52.0 [42.0–62.1]	*164*	68.6 [60.9–76.3]	*108*	81.0[71.6–90.4]	*47*	115.9[95.0–136.9]	*34*	129.2 [107.0–151.4]	<0.001
**ALT (IU/L)**	*46*	80.4 [59.1–101.7]	*164*	98.2 [85.3–111.1]	*108*	116.9 [98.9–134.9]	*47*	152.6 [122.2–183.]	*34*	147.4[116.0–178.7]	<0.001
**ALP (IU/L)**	*46*	81.4 [74.3–88.5]	*164*	79.7[75.7–83.7]	*108*	81.4 [77.1–85.6]	*47*	98.8 [88.9–108.7]	*34*	98.0 [86.0–110.0]	<0.001
**Albumin (IU/L)**	*46*	43.1 [42.2–44.0]	*164*	43.5 [43.1–43.9]	*108*	43.1 [42.5–43.7]	*47*	42.9 [42.2–43.7]	*34*	40.6 [39.1–42.2]	<0.001
**Bilirubin (μmol/L)**	*46*	10.8 [9.3–12.4]	*164*	11.4 [10.5–12.2]	*108*	10.9 [10.0–11.8]	*47*	13.0 [11.4–14.6]	*34*	15.3 [12.9–17.7]	0.001
**gGT (IU/L)**	*46*	58.5 [42.0–75.0]	*164*	69.5 [54.0–85.1]	*108*	83.1 [67.8–98.3]	*47*	124.7 [95.8–153.7]	*34*	117.5 [90.9–144.1]	<0.001
**Creatinine (μmol/L)**	*30*	78.6 [71.6–85.7]	*103*	77.3 [74.5–80.1]	*87*	75.5 [72.3–78.7]	*38*	73.4 [69.4–77.3]	*27*	73.3 [68.2–78.4]	0.473
**INR**	*48*	0.931 [0.907–0.955]	*165*	0.965 [0.954–0.976]	*105*	0.955 [0.943–0.968]	*48*	0.996 [0.977–1.015]	*33*	1.06 [1.02–1.09]	<0.001

CI, Confidence Interval; BMI, Body Mass Index; AST, Aspartate Transaminase; ALT, Alanine Transaminase; ALP, Alkaline Phosphatase; gGT, Gamma Glutamyltransferase; INR, International Normalized Ratio; n, number of observations; ANOVA, One-way analysis of variance.

### Relationship between the biomarkers and Metavir Fibrosis stages

**Fig 1:**
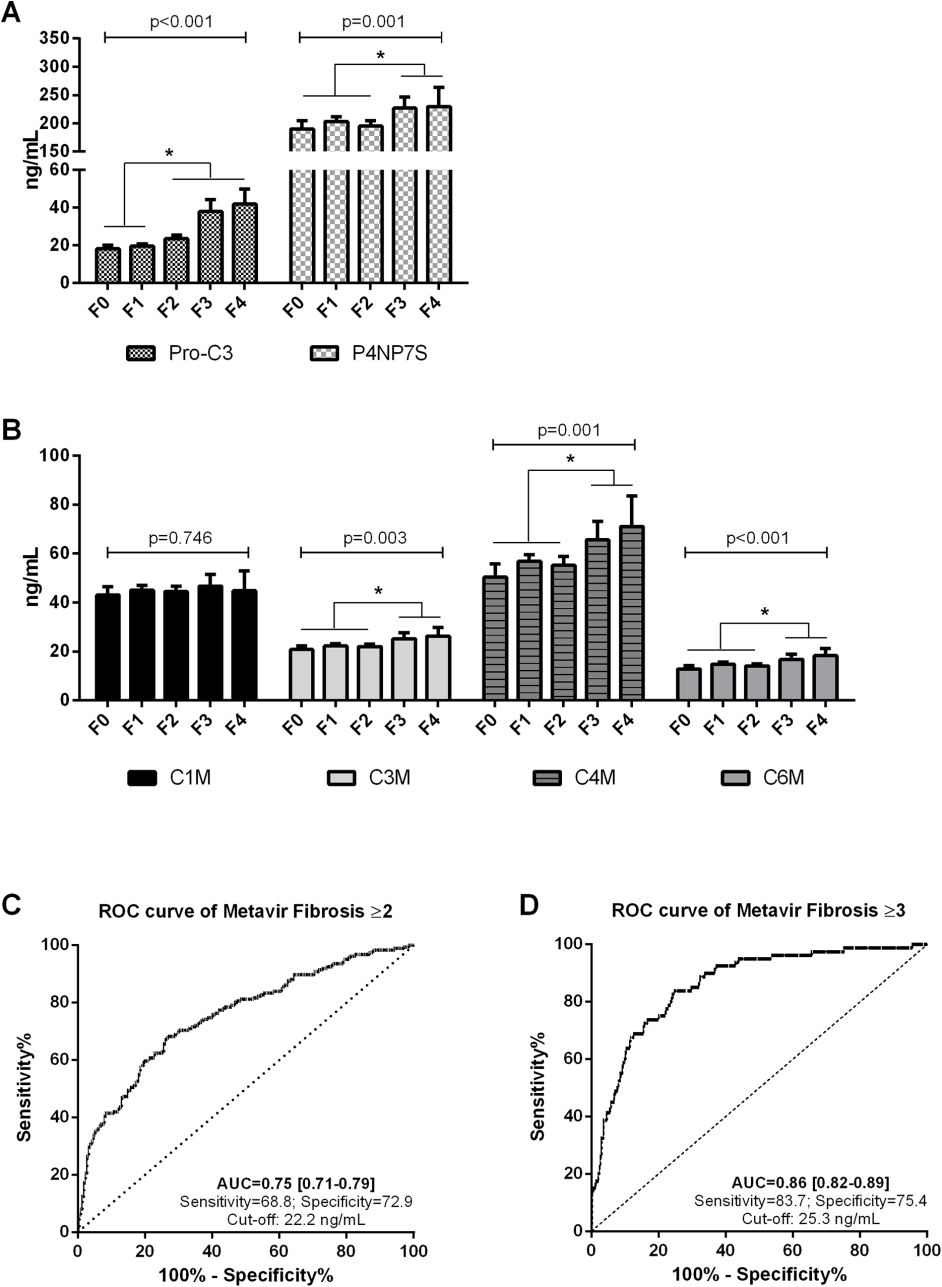
Diagnostic performance of individual Protein Fingerprint markers for detecting HCV patients. **A)** Plasma levels of Pro-C3 and P4NP7S, **B)** Plasma C1M, C3M, C4M and C6M in chronic HCV patients stratified according to Metavir F stages. F0 n = 47, F1 n = 167, F2 n = 107, F3 n = 45 and F4 n = 35. **C)** Receiver operating characteristic curve (ROC) analysis for the performance of Pro-C3 in distinguishing between F0-F1 (n = 214) and significant fibrosis (≥F2) (n = 186) in chronic HCV patients; **D)** ROC analysis for the performance of Pro-C3 in distinguishing between F0-F2 (n = 320) and significant fibrosis (≥F3) (n = 80) in chronic HCV patients. Data are shown as geometric mean (95%CI). Asterisks indicate statistical significance indicated by bars. *P<0.05.

Mean plasma levels of the Protein Fingerprint markers were stratified according to Metavir fibrosis stages (Fig 1A and 1B). All markers except C1M showed significant differences between the groups (p<0.01). Only Pro-C3 was significantly elevated in patients with significant fibrosis (≥F2) compared to F0-F1 (p<0.05), while the markers C3M, C4M, C6M, and P4NP7S were significantly elevated in patients with advanced fibrosis (≥F3) compared to F0-F2 (p<0.05).

Further, ALT was significantly elevated in patients with ≥F2 compared to F0 (p<0.05), whereas AST was the only marker significantly increased in patients with ≥F1 compared to patients without fibrosis (F = 0) (p<0.05) (data not shown).

The area under the ROC curves were used to evaluate the diagnostic values for detecting F≥2 and F≥3 (Table 2). The best marker was Pro-C3 with AUCs = 0.75 for detecting F≥2 (Fig 1C) and AUCs = 0.86 for detecting F≥3 (Fig 1D). A pair-wise comparison of AUCs showed that Pro-C3 was significantly better at detecting F≥2 and F≥3 compared to the other markers (P<0.0001).

**Table 2:** Diagnostic performances of Protein Fingerprint markers for the detection of significant (≥F2) and advanced (≥F3) fibrosis.

Fibrosis stage	Marker	Sensitivity (%)	Specificity (%)	AUC [95%CI]	*P*-Value
**≥F2 (Prevalence for ≥F2: 53%)**	**C3M**	19.9	93.0	0.56 [0.51–0.61]	0.031
	**C4M**	48.4	64.0	0.56 [0.51–0.61]	0.031
	**C6M**	51.9	58.9	0.55 [0.50–0.60]	0.068
	**Pro-C3**	68.4	72.9	0.75 [0.71–0.79]*	<0.0001
	**P4NP7S**	55.6	53.7	0.54 [0.49–0.59]	0.210
**≥F3 (Prevalence for ≥F3: 20%)**	**C3M**	45.0	77.3	0.64 [0.59–0.68]	0.0003
	**C4M**	42.5	80.4	0.64 [0.60–0.69]	0.0001
	**C6M**	66.2	57.3	0.64 [0.59–0.69]	0.0001
	**Pro-C3**	83.7	75.4	0.86 [0.82–0.89]*	<0.0001
	**P4NP7S**	68.7	59.8	0.63 [0.58–0.68]	0.003

*) AUC significantly different from C3M, C4M, C6M and P4NP7S for detecting ≥F2 and ≥F3

### Improved detection of fibrosis by multimarker models

To improve the diagnostic power for detection of liver fibrosis, we performed multiple ordered logistic regression analyses with Metavir Fibrosis score as dependent variable and protein fingerprint markers as well as other continuous variables as independent variables. Model 1 was built on the hypothesis that structural changes of ECM as well as liver tests are associated with fibrosis. In this model the parameters Pro-C3, C4M, AST, and ALT were significantly associated with Metavir Fibrosis score (p<0.0001–0.026) resulting in an algorithm with the following equation: -0.72 x logALT + 1.69 x logAST + 3.81 x logProC3 + 1.53 x logC4M (r = 0.54; p<0.0001).

Model 2 was built on the hypothesis that a combination of structural changes and demographic/clinical risk factors might improve liver fibrosis staging. Thus model 2 included Pro-C3, C4M, age, BMI, and gender resulting in an algorithm with the following equation: -0.18 x logBMI + 0.34 x gender + 5 x logAge + 4.87 x logProC3 + 2.11 x logC4M (r = 0.59; p<0.0001).

**Fig 2:**
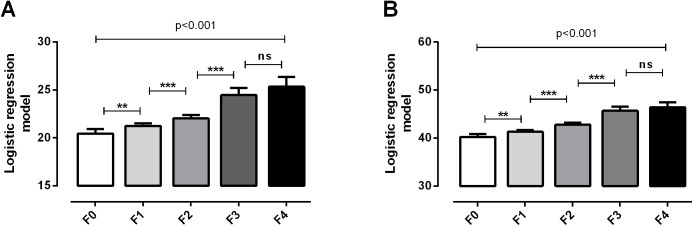
Multiple ordered logistic regression models for the detection of fibrosis stratified according to Metavir F stages. **A)** Model 1 combining Pro-C3, C4M, AST, and ALT; **B)** Model 2 combining Pro-C3, C4M, age, BMI, and gender. Data are shown as geometric mean (95%CI) calculated from the algorithms. Asterisks indicate statistical significance indicated by bars. *P<0.05, **P<0.01, ***P<0.001.

Both models detected patients with ≥F2 and ≥F3 with AUCs = 0.76–0.88 (Table 3). Furthermore, Model 1 and 2 predicted ≥F1 compared to F0 (Fig 2A and 2B) with AUCs of 0.74 [95% CI 0.70–0.79], p<0.0001 and 0.75 [95% CI 0.71–0.80], p<0.0001, respectively.

**Table 3:** Diagnostic performance of combination models for the detection of significant (≥F2) and advanced (≥F3) fibrosis.

Fibrosis stage	Marker	Sensitivity (%)	Specificity (%)	AUC [95%CI]	*P*-Value
**≥F2 (Prevalence for ≥F2: 53%)**	**FIB-4**	69.6	72.1	0.76 [0.72–0.80]	<0.0001
	**APRI**	56.8	82.2	0.74 [0.69–0.78]	<0.0001
	**Model 1**	63.5	78.4	0.76 [0.72–0.81]	<0.0001
	**Model 2**	69.8	78.7	0.80 [0.76–0.84]*	<0.0001
**≥F3 (Prevalence for ≥F3: 20%)**	**FIB-4**	76.2	76.6	0.84 [0.80–0.86]	<0.0001
	**APRI**	76.2	75.7	0.82 [0.78–0.86]	<0.0001
	**Model 1**	89.0	76.0	0.88 [0.84–0.91]†	<0.0001
	**Model 2**	87.0	76.5	0.88 [0.84–0.91]	<0.0001

*) AUC significantly different from Pro-C3 and APRI for detecting ≥F2

†) AUC significantly different from APRI for detecting ≥F3

A pair-wise comparison showed that the established FIB-4 index provided similar diagnostic power as Model 1 and 2 for detecting patients with ≥F2 and ≥F3. Compared to APRI, Model 2 was significantly better for detecting ≥F2 (p = 0.046), while Model 1 was significantly better for detecting F≥3 (p = 0.023) (Table 3). Furthermore, Model 2 was significantly better at detecting ≥F2 than Pro-C3 as single marker (p = 0.020).

## Discussion

In the present study we investigated novel Protein Fingerprint biomarkers for liver fibrosis staging in HCV patients. The principal findings were: 1) Pro-C3 provided clinically relevant diagnostic accuracy as single marker of significant (≥F2) and advanced (≥F3) liver fibrosis; 2) a combination of the markers Pro-C3 and C4M with age, BMI, and gender improved the diagnostic accuracy; 3) a multimarker model was able to separate F0 from F1.

Non-invasive markers such as serum markers or FibroScan may be used to aid and/or replace the need for liver biopsy to stage liver fibrosis. Most non-invasive markers are able to diagnose extreme stages of fibrosis however the “grey area” between Metavir F1 and F2 still remains a clinical challenge [26]. We have previously shown that Pro-C3 is different from the conventional marker PIIINP [16] and in the present study Pro-C3 was able to identify patients with clinically significant fibrosis (≥F2) with AUC = 0.75. Pro-C3 measures true formation of type III collagen as the antibody is directed against the cleavage site of the N-terminal propeptide from the helix. In contrast, the PIIINP antibody is not specific for cleavage of the propeptide and therefore measures both degradation and formation products reflecting fibrolysis and/or fibrogenesis. Measurement of PIIINP has been widely evaluated in clinical settings as a diagnostic marker of severe liver fibrosis with AUCs from 0.73–0.87 [27–29]. However the use of PIIINP for detecting both significant and severe fibrosis shown by Leroy *et al* (AUC = 0.77 and 0.88) and Saitou *et al* (AUC = 0.75 and 0.79) [30, 31] is similar to our data. An important confounder is the observation that PIIINP is increased in patients receiving antiviral therapy [32]. In our study none of the patients received antiviral therapy prior to inclusion, thus further studies are needed to investigate the confounding effects of therapy on Pro-C3.

Interestingly, *formation* of type III collagen was up-regulated earlier in patients with chronic HCV than *degradation* of type III collagen reflected by a significant increase in Pro-C3 in patients with Metavir F≥2 compared to C3M, which was not significantly increased until Metavir F≥3. Similarly, the basement membrane remodeling markers, P4NP7S and C4M, were not up-regulated until advanced fibrosis stages, indicating that development of a basement membrane-like structure in the Space of Disse occurs later in fibrosis development than remodeling of interstitial matrix, reflected by C3M and Pro-C3. By contrast, C1M did not show any relevance to liver fibrosis in these patients. This might indicate that degradation of type I collagen does not occur until cirrhosis which is supported by our previous study showing significantly up-regulated C1M in cirrhotic patients with portal hypertension [33]. Furthermore, we have previously shown that Pro-C3 was able to segregate individual fibrosis stages as well as predicting change in fibrosis score after 52 weeks in patients with chronic HCV while C3M was unable to predict fibrosis progression [34]. This indicates that measurement of different sub-pools of the same protein provides different diagnostic information as the tissue turnover balance between formation and degradation is what actually defines fibrosis. Intracellular uptake of degraded collagen fragments may prevent further inflammation of the tissue, as cleaved collagen fragments can induce acute inflammation through neutrophil recruitment [35, 36]. This phenomenon may be reflected by the levels of C3M, C4M, and C6M in this study. In the early stages of fibrosis with little ECM accumulation, the cellular uptake of collagen fragments may be more efficient than in the advanced fibrosis stages with more inflammation, ECM accumulation, and MMP activity. This suggests that the protein fingerprint markers may not only be fibrosis markers but also activity markers.

Several composite algorithms of non-invasive tests have been evaluated for the diagnosis of fibrosis in HCV patients. These tests are either based on serological markers alone or in combination with FibroScan. We proposed two models for diagnosing significant fibrosis in HCV infection. Model 1 correlated to Metavir Fibrosis stages however the diagnostic performance was only slightly better than using Pro-C3 as an individual marker. Model 2 was significantly better than Pro-C3 and APRI for detecting ≥F2 with an AUC = 0.80. Several studies have evaluated the diagnostic performances of APRI and FIB-4 as non-invasive algorithms for detecting significant and/or advanced fibrosis [37–40]. They all found similar results as us except Wai *et al*. who found a mean APRI AUC for significant fibrosis of 0.84 [22]. Common for the studies are, that FIB-4 shows somewhat better diagnostic accuracy than APRI however with overlapping confidence interval and therefore not significantly different. The ELF test showed similar AUCs for detecting significant and advanced fibrosis ranging from 0.74–0.87 and 0.84–0.89, respectively [9], which is similar to the diagnostic performance of our proposed Models 1 and 2. Interestingly, our models were able to differentiate early fibrosis stages, i.e. Metavir F0 and F1 with AUCs = 0.76 and sensitivities around 80%. The low specificity of approximately 60% in these models for differentiating F0 from F1 might be due to inter-/intra-observer variability in the liver biopsy, which was used as reference as it is well known that staging of fibrosis can vary within 1–2 fibrosis stages [3]. Further studies are needed to confirm the diagnostic value of our models to diagnose early fibrosis stages.

The major strength of the study is the large group of treatment naïve well-characterized biopsy proven HCV patients covering the entire spectrum. A limitation of the study is the missing comparison to novel elastography techniques, such as FibroScan or MR elastography, as they fulfill many of the requirements of an ideal non-invasive marker of fibrosis. However elastographic techniques carry some limitations as well, including requirement for expensive equipment, considerable expertise, and lack of standardized cut-off for diagnosis of fibrosis stages [41]. Thus the protein fingerprint markers may be of more clinical relevance in the general clinical practices when monitoring treatment deferred patients to reconsider indication of treatment and to discuss new therapies as they emerge. Despite the fact that the protein fingerprint markers as well as the two models proposed in this study detect significant and advanced fibrosis with similar accuracy as the indirect markers APRI and FIB-4, the fingerprint markers may still be of clinical relevance as they reflect ECM turnover and processes related to fibrogenesis and not liver function as APRI and FIB-4. Another limitation is the cross-sectional design in which prognostic evaluation of the markers is not possible, as well as the need for replication in other cohorts.

In conclusion we assessed a panel of specific protein fingerprint markers for detection of clinical significant and advanced fibrosis in patients with chronic HCV. Pro-C3 proved useful as single diagnostic marker; however the multimarker models improved the diagnostic value for detecting significant and advanced fibrosis. Prospective studies are needed to evaluate the prognostic utility of the Protein Fingerprint markers for deciding on earlier antiviral therapy and as efficacy markers.
